# Period 2 is essential to maintain early endothelial progenitor cell function *in vitro* and angiogenesis after myocardial infarction in mice

**DOI:** 10.1111/jcmm.12241

**Published:** 2014-03-13

**Authors:** Yuan-Yuan Sun, Wen-Wu Bai, Bo Wang, Xiao-Ting Lu, Yi-Fan Xing, Wen Cheng, Xiao-Qiong Liu, Yu-Xia Zhao

**Affiliations:** a Key Laboratory of Cardiovascular Remodeling and Function Research, Qilu Hospital, Shandong UniversityJinan, Shandong, China; b Department of Traditional Chinese Medicine, Qilu Hospital, Shandong UniversityJinan, Shandong, China

**Keywords:** period 2, endothelial progenitor cells, myocardial infarction, angiogenesis

## Abstract

Cellular therapeutic neovascularization has been successfully performed in clinical trials for patients with ischaemia diseases. Despite the vast knowledge of cardiovascular disease and circadian biology, the role of the circadian clock in regulating angiogenesis in myocardial infarction (MI) remains poorly understood. In this study, we aimed to investigate the role and underlying mechanisms of Period 2 (Per2) in endothelial progenitor cell (EPC) function. Flow cytometry revealed lower circulating EPC proportion in per2^−/−^ than in wild-type (WT) mice. PER2 was abundantly expressed in early EPCs in mice. *In vitro*, EPCs from per2^−/−^ mice showed impaired proliferation, migration, tube formation and adhesion. Western blot analysis demonstrated inhibited PI3k/Akt/FoxO signalling and reduced C-X-C chemokine receptor type 4 (CXCR4) protein level in EPCs of per2^−/−^ mice. The impaired proliferation was blocked by activated PI3K/Akt/FoxO signalling. Direct interaction of CXCR4 and PER2 was detected in WT EPCs. To further study the effect of per2 on *in vivo* EPC survival and angiogenesis, we injected saline or DiI-labelled WT or per2^−/−^ EPC intramyocardially into mice with induced MI. Per2^−/−^ reduced the retention of transplanted EPCs in the myocardium, which was associated with significantly reduced DiI expression in the myocardium of MI mice. Decreased angiogenesis in the myocardium of per2^−/−^ EPC-treated mice coincided with decreased LV function and increased infarct size in the myocardium. Per2 may be a key factor in maintaining EPC function *in vitro* and in therapeutic angiogenesis *in vivo*.

## Introduction

Revascularization of ischaemic tissue is thought to occur through the migration and proliferation of mature endothelial cells in nearby tissues. Over the last several years, therapy with endothelial progenitor cells (EPCs) has been considered a promising therapy for myocardial infarction (MI). Endothelial progenitor cells circulate in the blood and home to sites of injured vascular or tissue, thus contributing significantly to both re-endothelialization and neoangiogenesis in the embryonic period or adulthood [1–4].

Endothelial progenitor cells were discovered and identified in 1997 by Asahara *et al*. [5] on the basis of vascular endothelial growth factor receptor-2 (VEGFR2 also known as KDR in humans or Flk1 in mice) and CD34 co-expression. Increased EPC number in peripheral blood enhances the repair of injured arteries and decreased EPC number predicts future cardiovascular events [6,7]. Improved mobilization of bone-marrow EPCs and their function can equally benefit the formation of coronary collateral circulation. Transplantation of modified EPCs into infarcted mouse hearts improved left ventricular (LV) function [8] by decreasing lymph angiogenesis [9] and increasing vasculogenesis and cardiomyogenesis [10]. However, despite the effectiveness of EPCs therapy in many studies, the benefit is limited in part by low survival and homing of transplanted EPCs [11]. Despite the vast knowledge of angiogenesis and MI, the role of the circadian clock in regulating angiogenesis remains poorly understood.

Period 2 (Per2), one of the rhythm genes, plays an important role in cardiovascular disease [12,13]. Per2 expresses in human bone-marrow CD34+ cells, human peripheral leucocytes and mouse bone marrow [14–16]. Per2 regulates the release of haematopoietic stem cells *via* circadian oscillations [17]. Investigators found no significant differences in EPC levels between wild-type (WT) and per2^m/m^ mice [18], and we observed EPC levels in both WT and per2^−/−^ mice at different times in the PER2 circadian rhythm. Per2 mutant mice showed impaired endothelial function, increased vascular senescence and decreased angiogenesis [18–20]. Per2^m/m^ mice in an ischaemia model showed decreased mobilization of bone-marrow EPCs [18]. However, improvement of cardiac function after MI is determined by the number of mobilized bone-marrow EPCs and function of transplanted cells [21–23].

The alteration in EPC function, the possible mechanism and angiogenesis after MI by Per2 knockout has not been investigated. Here, we aimed to investigate the effect of Per2 on circulating EPC levels and bone-marrow EPC function and the possible mechanism. We further investigated the importance of Per2 in regulating EPCs angiogenesis *in vivo* in a mouse model of MI.

## Materials and methods

### Animals

We obtained male C57BL/6 mice (8–12 weeks old) weighing 25–30 g from Vital River (Beijing, China). Per2^−/−^ mice were obtained from the Model Animal Research Center, Nanjing University (Nanjing, China), and backcrossed for more than 10 generations onto a C57BL/6 inbred background. All animal studies were carried out at the Animal Care Center of the Key Laboratory of Cardiovascular Remodeling and Function Research, Shandong University (Shandong, China). The experiment followed the Animal Management Rule of the Ministry of Public Health, People*s Republic of China (document no. 55, 2001), and the experimental protocol was approved by the Animal Care Committee of Shandong University.

Mice were acclimatized in the same room with a 12-h/12-h light–dark cycle for at least 2 weeks before experiments. Mice were anesthetized with sodium pentobarbital (50 mg/kg, intraperitoneally [i.p.]) and MI was induced by ligation of the left anterior descending coronary artery as described [24]. Mice were divided into three groups for treatment: intramyocardial injection of PBS, 5 × 10^5^ DiI-labelled WT or per2^−/−^ mouse bone-marrow EPCs [25] (see description of EPCs creation below) in a total volume of 30 μl at four sites (up, down, left and right) in the peri-infarct area immediately after surgery. Before transplantation, EPCs in suspension were washed with PBS and incubated with DiI (Sigma-Aldrich, St. Louis, MO, USA) at 2.5 μg/ml PBS for 5 min. at 37°C and 15 min. at 4°C. After two washes with PBS, cells were resuspended in PBS. Mice were killed 4 weeks after MI.

### Flow cytometry

Before being killed, WT and per2^−/−^ mice were anesthetized with sodium pentobarbital (50 mg/kg, i.p.) at 6:00, 12:00 and 18:00 (at 6:00, the light was turned on, and at 18:00, the light was turned off). For each time, six mice were killed. After extraction of peripheral blood, femurs and tibias were separated and placed in culture medium. Then, 200 μl blood was incubated with FITC-conjugated antibody for mouse CD34 and APC-conjugated antibody for mouse Flk1 (eBioscience, San Diego, CA, USA) at room temperature for 90 min. according to the manufacturer*s instructions. FITC- or APC-conjugated isotype IgG (eBioscience) was used as a control. Erythrocytes were lysed in lysis buffer (Solarbio, Beijing, China), centrifuged, and the supernatant was discarded. Cells were washed and resuspended in 1× PBS. Proportion of FITC-CD34+ and APC-Flk1+ cells was quantified by use of FACSCAlibur and Cell-Quest software (BD Biosciences, San Jose, CA, USA). In some experiments, PE-conjugated antibody against mouse CD45 and PE-conjugated isotype IgG were used.

### Bone-marrow EPC culture and identification

Bone-marrow-derived mononuclear cells were isolated by density gradient centrifugation at 2000 × *g* for 20 min. After three rinses, cells were seeded on cell-culture dishes coated with 0.1% mouse vitronectin (Sigma-Aldrich) with EBM-2 [Lonza, Walkersvile, MD, USA; basal medium with hydrocortisone, hFGF-B, VEGF, R3-IGF-1, ascorbic acid, hEGF, GA-100, heparin and 5% fetal bovine serum (FBS)]. After 4 days in culture, non-adherent cells were removed by washing with PBS; adherent cells were cultured for three additional days, and medium was changed every other day. On day 7, cells were co-incubated with 2.4 μg/ml 1,1V-dioctadecyl-3,3,3V,3-tetramethyl-indocarbocyanine perchlorate-labelled acetylated low-density lipoprotein (DiI-acLDL; Invitrogen, Carlsbad, CA, USA) for 1 hr, then counterstained with fluorescein isothiocyanate-labelled lectin from Ulex europaeus (FITC-UEA-1 lectin, Sigma-Aldrich) 10 μg/ml for 1 hr; nuclei were stained with 4′,6-diamino-2-phenyl indole (DAPI; Beyotime, Nantong, China) and cells were viewed by laser scanning confocal microscopy (LSM710; Zeiss, Jena, Germany) [26,25]. Cells double stained with the two markers were defined as EPCs and further characterized by flow cytometry with stem and endothelial cell markers (CD34, Flk1; CD45; eBioscience) as described previously [27].

### Quantitative RT-PCR

Total cellular RNA was obtained by use of TRIZOL reagent (Invitrogen), quantified by spectrophotometry and reverse transcribed by the use of the M-MLV Reverse Transcriptase System (Osaka) with oligo-dT primers. Per2 mRNA expression was examined by real-time RT-PCR with the SYBR Green Real-time PCR Master Mix (Toyobo, Osaka, Japan) and MYIQTM Single Color Real-Time PCR Detection System (Bio-Rad, Hercules, CA, USA). The primer sequences (from Genbank, AF 035830) were for PER2, forward, 5′-CTCCAGCGGAAACGAGAACTG-3′, and reverse, 5′-TTGGCAGACTGCTCACTACTG-3′). Actin (forward, 5′-GTGACGTTGACATCCGTAAAGA-3′, and reverse, GCCGGACTCATCGTACTCC-3′) was used as an internal control. Data were analysed by the 2^−ΔΔCT^ method.

### Immunofluorescence analysis

Per2 protein expression was evaluated by immunofluorescence staining. Endothelial progenitor cells were rinsed with PBS and fixed with 4% paraformaldehyde (Sigma-Aldrich) in PBS for 30 min., then rinsed three times with PBS, incubated with 0.3% Triton X-100 (Sigma-Aldrich) in PBS for 5 min., washed twice with PBS and incubated overnight at 4°C with primary antibodies diluted with 1% FBS in PBS. After three washes with PBS, cells were incubated with secondary antibodies for 1 hr at 37°C, then rinsed three times with PBS, stained with DAPI to visualize cell nuclei, rinsed three times with PBS, dried and viewed by fluorescence microscopy at 400× magnification.

### EPC proliferation assay

Endothelial progenitor cell proliferation was evaluated by use of the cell counting kit-8 (CCK8; BestBio, Shanghai, China) [28]. In brief, after incubation with 1% serum medium for 24 hrs, EPCs (day 7) were re-seeded on 96-well plates (1 × 10^4^/well) coated with 0.1% mouse vitronectin. In some experiments, cells were incubated with IGF-1 [activator of phosphoinositide 3-kinase (PI3K); 20 ng/ml; R&D, Wiesbaden, Germany] [29] or LY294002 (inhibitor of PI3K; 10 μM, Selleck, Houston, MN, USA). After 24 hrs in culture, CCK-8 solution was added to each well for 1 hr at 37°C. Absorbance was measured at 450 nm by use of a microplate reader (Varioskan Flash, Dreieich, Thermo Fisher, Germany). Each test was repeated three times.

### EPC migration analysis

Endothelial progenitor cell migration was evaluated by a modified Boyden chamber (Costar, Tewksbury, MA, USA) assay [30]. After incubation with 1% serum medium for 24 hrs, EPCs (day 7) were placed in the upper chamber (2 × 10^4^ cells/well) with 200 μl EBM-2 medium, then the chamber was placed in a 24-well culture dish containing 500 μl EBM-2 with VEGF (50 ng/ml) [31] and 5% FBS. In some experiments, cells were incubated with IGF-1 (20 ng/ml; R&D). After incubation for 12 hrs at 37°C, membranes were washed briefly with PBS. The upper side of the membrane was fixed with 4% paraformaldehyde (Sigma-Aldrich) in PBS for 30 min., then wiped gently with a cotton ball to remove non-migratory cells and stained with 1% crystal violet in 2% ethanol. Data are presented as mean number of migrating cells in five randomly selected fields at 200× magnification in every membrane. All groups were studied in triplicate.

### Matrigel tube formation assay

Matrigel matrix (BD Biosciences) was used to evaluate capillary-like tube formation of EPCs. An amount of 50 μl matrigel was placed in 96-well plates to solidify. Endothelial progenitor cells (1 × 10^4^/well) were resuspended in 50 μl basic EGM-2 and plated on matrigel for 8 hrs at 37°C. In some experiments, cells were incubated with IGF-1 (20 ng/ml; R&D). The mean tube length was calculated in five randomly chosen fields at 100× from each well after being photographed.

### Adhesion assay

Endothelial progenitor cells from WT and per2^−/−^ mouse bone marrow were re-seeded on 96-well plates (1 × 10^4^/well) coated with 0.1% mouse vitronectin for assay of adhesive activity. In some experiments, cells were treated with IGF-1 (20 ng/ml; R&D). After 1 hr, non-adherent cells were washed off with PBS and adhesive cells were fixed with 4% paraformaldehyde (Sigma-Aldrich) in PBS for 30 min., then stained with 1% crystal violet in 2% ethanol [31]. Data are presented as mean number of adhered cells in five randomly selected fields at 200× magnification in every well. All groups were studied in triplicate.

### Western blot analysis

After treatment, adherent cells were washed twice with PBS, lysed in lysis buffer (TBD sciences) on ice for 30 min., then centrifuged at 12,000 × g for 15 min. at 4°C. The supernatant (cytosolic fraction) was removed, and protein concentrations were measured by BCA assay (Beyotime) with β-actin (ZSGB-BIO) as a loading control. Membranes with protein were incubated with primary antibodies for CXCR4 (Abcam, San Francisco, CA, USA), PI3k p110α (Cell Signalling Technology, Danvers, MA, USA), and total and phosphorylated forms of Akt (Cell Signaling Technology) and total and phosphorylated forms of FoxO3a (Abcam) at 4°C overnight, washed, then incubated for 30 min. with horseradish peroxidase-conjugated secondary anti-IgG antibody. The membranes were washed three times for 5 min. each and developed with Super Signal chemiluminescent substrate (Millipore, Schwalbach, Germany).

### Co-IP detection of protein interaction

Cell lysates were prepared as described previously [32]. Co-IP involved rabbit anti-per2 or CXCR4 antibody (Santa Cruz Biotechnology Inc., San Francisco, CA, USA) to bind CXCR4 or per2, followed by incubation with protein A and G plus-agarose beads (Santa Cruz Biotechnology). The protein interacting with per2 or CXCR4 was detected by western blot analysis using CXCR4 or per2 antibodies.

### Echocardiography

Transthoracic echocardiography was performed to evaluate LV function in mice before and 28 days after MI by use of high-frequency duplex ultrasonic cardiography (Visual Sonics Vevo 770, Toronto, ON, Canada) and a transducer (RMVTM Scan Head 710B-048, Visual Sonics, Toronto, ON, Canada). With mice under general anaesthesia with isoflurane, LV diastolic and systolic internal dimensions (LVIDD and LVIDS respectively) were measured at the mid-papillary muscle level. The system calculated the LV ejection fraction (EF) and LV fractional shortening (FS).

### Histology and immunochemistry

Mice were killed at 28 days after MI, and hearts were rapidly excised after saline perfusion. We selected six mice in each group to obtain frozen tissue sections. Cardiac tissues were cut into 5-μm slices, and DiI expression was observed by fluorescence microscopy. Cardiac tissues of the remaining mice were fixed with 4% paraformaldehyde for 72 hrs and embedded in paraffin, then cut into 5-μm sections for analysis. Sections underwent immunohistochemical analysis or Masson*s trichrome staining. Tissue sections were deparaffinized, and endogenous peroxidase was inhibited with 3% H_2_O_2_. After being rinsed in PBS, sections were blocked with 5% goat serum in PBS, then incubated with rabbit anti-α smooth muscle actin (α-SMA; Abcam) for determining arteriolar density and anti-CD31 (R&D) for microvascular density in infarcted hearts. The reaction product was visualized by staining with 3, 3-diaminobenzidine (DAB, Vector Laboratories, Burlingame, CA, USA). Arteriolar and capillary density was measured in the peri-infarct zone in five randomly selected fields per section at 400× magnification; the mean number of vessels was used for assessment of vascular density.

The infarct scar size was measured by Masson*s trichrome staining by computer morphometry with Adobe Photoshop software and expressed as a percentage of the whole left ventricle mid-ventricular section.

### Apoptosis assay

Cell apoptosis was assessed by use of a TUNEL kit (Millipore). TUNEL-positive cells (DAB+) were counted in five randomly selected fields of a section until 100 nuclei had been calculated. Every section was analysed three times, and data are shown as the mean.

### Statistical analysis

Data are described as mean ± SEM. Comparisons between two groups involved Student*s *t*-test and multiple groups anova with Bonferroni*s correction. SPSS v16.0 (SPSS Inc., Chicago, IL, USA) was used for analysis. *P* < 0.05 was considered statistically significant.

## Results

### Per2^−/−^ mice showed altered EPC number

**Fig. 1:**
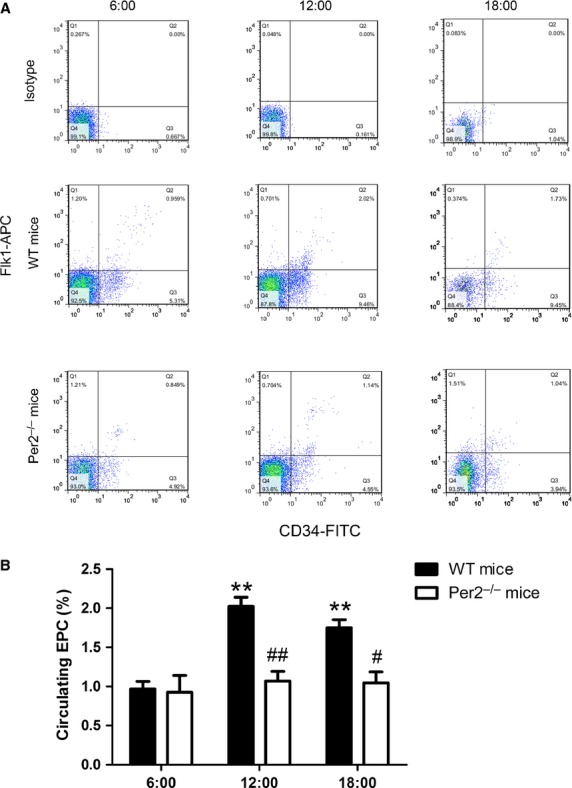
Differential proportion of circulating endothelial progenitor cells (EPCs) in mice. (**A**) Representative flow cytometry analysis of circulating EPC proportion in wild-type (WT) and Period 2-knockout (per2^−/−^) mice at different times. (**B**) Quantification. Data are representative of EPCs from three time points (**P < 0.01 *versus* 6:00, #*P* < 0.05, ##*P* < 0.01 *versus* WT mice at the same time; *n* = 6 per group).

Wild-type but not per2^−/−^ mice showed variation in circulating EPCs proportion (CD34+ Flk1+ cells) at different times (Fig. 1A). The proportion of circulating EPCs was greater in WT mice than in per2^−/−^ mice at 12:00 and 18:00 (Fig. 1A and B). Thus, Per2 may play an important role in regulating circulating EPC number.

### PER2 expresses in cultured EPCs

**Fig. 2:**
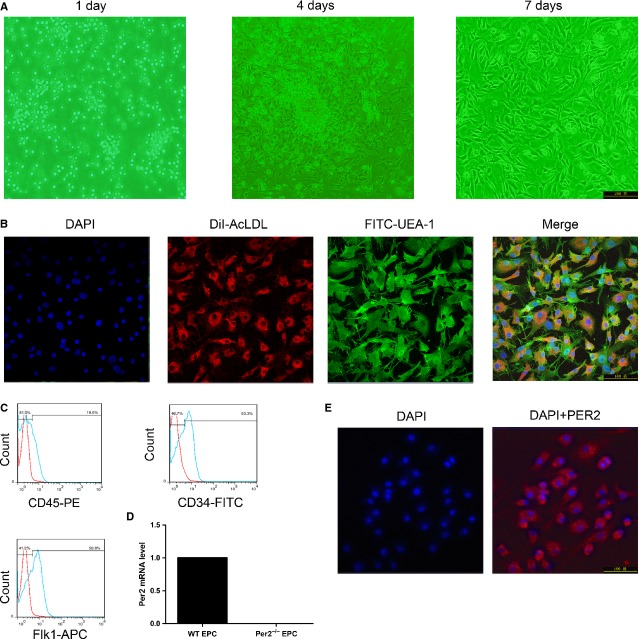
Characterization of cultured bone-marrow-derived endothelial progenitor cells (EPCs). (**A**) EPCs cultured on days 1, 4 and 7. (**B**) EPCs were identified by DiI-acLDL, FITC-UEA-1 and DAPI staining. Cells positive for all three dyes were defined as EPCs. (**C**) Flow cytometry analysis of CD34 or CD45 or Flk1 expression on EPCs. (**D**) RT-PCR analysis of per2 mRNA expression in cultured EPCs. (**F**) Immunofluorescence of PER2 expression in cultured EPCs.

Cells began to form clusters on day 4 of EPC culture (Fig. 2A). After 7 days of culture, >90% of cells were capable of cellular uptake of acLDL and UEA-1 lectin binding (Fig. 2B). More than half of cultured cells at 7–10 days were positive for the endothelial lineage marker Flk1 and stained positive for the progenitor cell marker CD34, although most cells did not express the hematopoietic lineage marker CD45 (Fig. 2C). These cells were identified as EPCs. To confirm whether Per2 expresses on cultured EPCs, PER2 mRNA and protein expression were detected in WT mouse bone-marrow EPCs by RT-PCR and immunofluorescence staining (Fig. 2D and E).

### Per2^−/−^ impairs proliferation, migration, tube formation and adhesion of EPCs

**Fig. 3:**
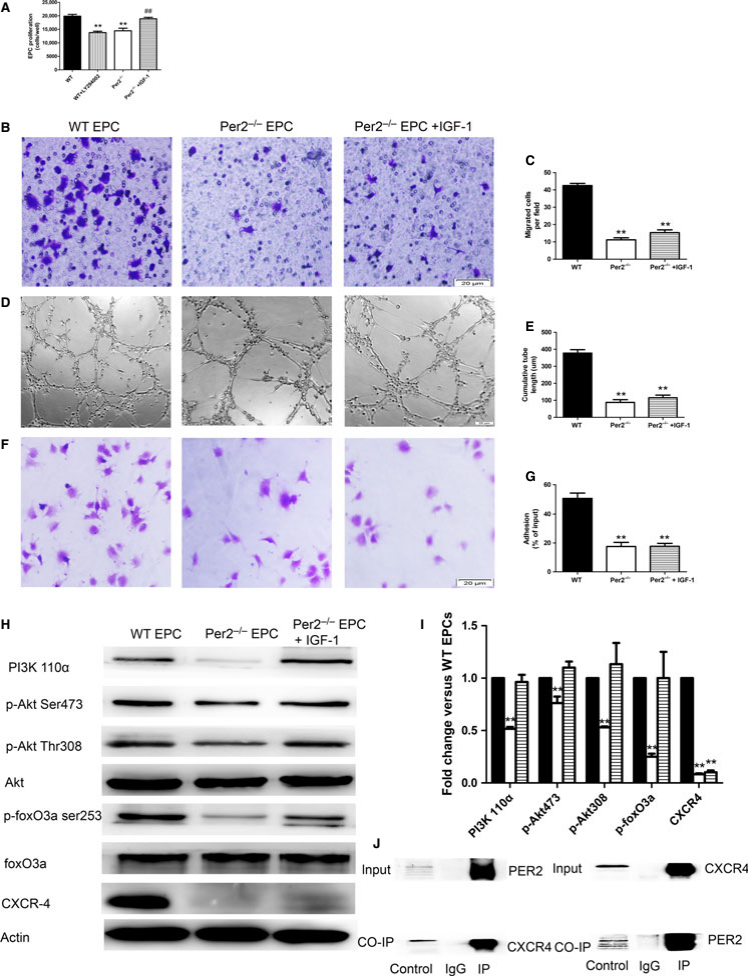
Effect and mechanism of per2 on endothelial progenitor cell (EPC) proliferation, migration, tube formation and adhesion. (**A**) EPC proliferation, (**B**) migration and (**C**) quantification, (**D**) tube formation and (**E**) quantification, and (**F**) adhesion and (**G**) quantification were impaired with per2 knockout. And, activation of PI3K with insulin-like growth factor 1 (IGF-1) rescued the impaired proliferation of per2^−/−^ EPCs. (**H** and **I**) Western blot analysis of PI3K/Akt/FoxO and CXCR4 expression. (**J**) Co-IP for direct interaction of PER2 and CXCR4 (*P < 0.05, **P < 0.01 *versus* wild-type, ##*P* < 0.05 *versus* per2^−/−^ *n* = 6).

To study the effect of Per2 on EPC function, we compared the function of bone-marrow EPCs from WT and per2^−/−^ mice *in vitro*. Endothelial progenitor cells from per2^−/−^ mice showed impaired proliferation, migration, tube formation and adhesion as compared with WT EPCs (Fig. 3A–G). Treating Per2^−/−^ EPCs with IGF-1 (activator of PI3K) rescues the impaired proliferation (Fig. 3A). LY294002 (inhibitor of PI3K) inhibited the proliferation of WT EPCs (Fig. 3A).

### Per2^−/−^ down-regulates PI3k/Akt/FoxO, CXCR4 in EPCs, with direct interaction of PER2 and CXCR4

Akt is central to cell proliferation signalling. The activation and inhibition of Akt depends on phosphorylation by PI3K at two critical regulatory sites: T308 within its kinase domain and S473 within a C-terminal hydrophobic motif. FoxO3a is an Akt down-regulated transcription factor. Compared with WT EPCs, per2^−/−^ EPCs showed lower phosphorylation of Akt Ser473 and Thr308, as well as FoxO3 (Fig. 3H and I). Phosphoinositide 3-kinase (p110α) protein expression was lower in per2^−/−^ than in WT EPCs; lower p-Akt level was because of inhibited PI3k p-110α activation. Phosphoinositide 3-kinase p-110α activated by IGF-1 increased p-Akt and FoxO3 levels (Fig. 3H). To investigate more mechanism of Per2 affecting EPC function, we tested CXCR4 in cultured EPCs. The expression of CXCR4 was reduced in per2^−/−^ mouse EPCs (Fig. 3H and I), and IGF-1 did not rescue its expression. To determine whether the two proteins were interacting, we performed Co-IP assay. In WT EPCs, PER2 and CXCR4 showed direct interaction (Fig. 3J).

### Per2^−/−^ decreased cardiac function and increased infarct size after MI

**Fig. 4:**
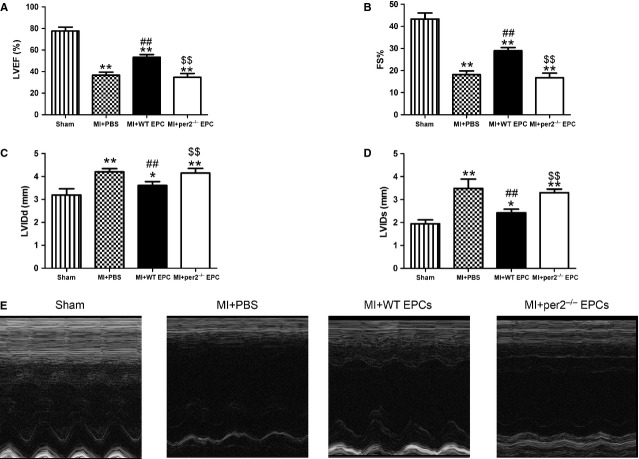
Effect of endothelial progenitor cells (EPCs) with or without per2 on mouse cardiac function 4 weeks after myocardial infarction (MI). (**A**) LV ejection fraction, (**B**) fractional shortening, (**C**) LV diastolic internal dimension and (**D**) LV systolic internal dimension. (**E**) Representative M-mode findings 4 weeks after MI (*P < 0.05, **P < 0.01 *versus* sham, ##*P* < 0.01 *versus* MI+PBS, $$*P* < 0.01 *versus* MI+wild-type EPCs; *n* = 6 per group).

**Fig. 5:**
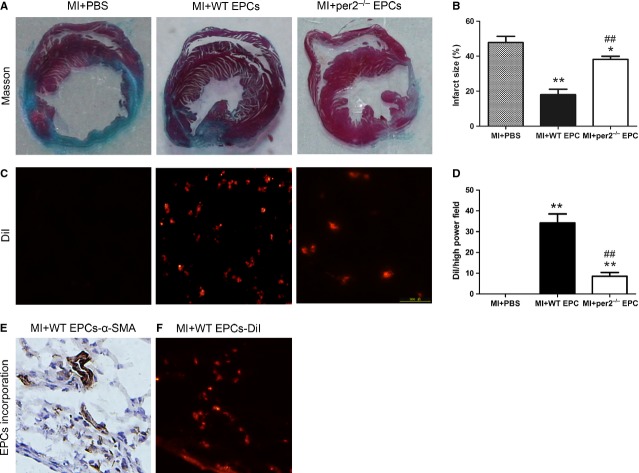
Effect of endothelial progenitor cells (EPCs) with or without per2 on mouse infarct area 4 weeks after myocardial infarction (MI). (**A**) Representative infarct area in the three groups and (**B**) quantification. (**C**) Representative expression of DiI-labelled EPCs in the myocardium and (**D**) quantification (*P < 0.05, **P < 0.01 *versus* MI+PBS, ##*P* < 0.01 *versus* MI+wild-type EPCs; *n* = 6). (**E**) α-Smooth muscle actin (α-SMA) expression in mouse cardiac tissues. (**F**) DiI-labelled EPC expression in similar locations.

Transthoracic echocardiographic examination was performed to evaluate cardiac function in mice after MI (Fig. 4A–E). At 4 weeks after injection, LVEF and LVFS were greater and LVIDD and LVIDS lower in mice with WT than with per2^−/−^ EPC and saline injection, with no difference between per2^−/−^ EPC and saline injection. Infarct size was larger with per2^−/−^ EPC and saline than with WT EPC injection (Fig. 5A and B). Thus, EPCs helped protect cardiac function and reduced infarct size after MI, which were reversed by knockout of Per2.

### Per2^−/−^ decreased survival and incorporation of injected EPCs after MI

To investigate the effect of Per2 on EPC function *in vivo*, DiI-labelled WT and per2^−/−^ EPCs cultured *in vitro* were injected into mouse myocardia after MI. At 4 weeks, the number of DiI-labelled per2^−/−^ EPCs was lower than that of WT EPCs in the MI myocardium (Fig. 5C and D). Wild-type but not per2^−/−^ EPCs showed typical EPCs incorporation into vasculature (Fig. 5E and F). Therefore, survival and incorporation into vasculature of EPCs was weakened by knockout of Per2 in the ischaemic myocardium of mice.

### Per2^−/−^ increased apoptosis after MI

**Fig. 6:**
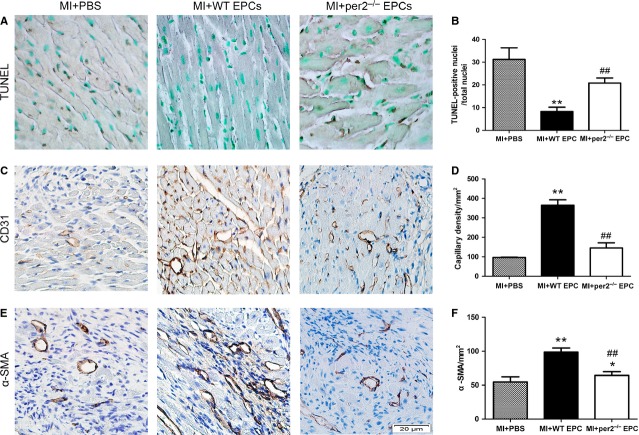
Effect of endothelial progenitor cells (EPCs) with or without per2 on apoptosis, capillary density and arteriole density 4 weeks after myocardial infarction (MI). Representative expression of (**A**) DAB+ nucleus (apoptotic cells), and (**B**) quantification (**C**) capillary density and, (**D**) quantification and (**E**) arteriole density and (**F**) quantification (*P < 0.05, **P < 0.01 *versus* MI+PBS, ##*P* < 0.01 *versus* MI+wild-type EPCs; *n* = 6 per group).

We assessed the survival of transplanted cells as well as the effect of cell transplantation on survival of resident myocytes in mice by apoptosis assay of mouse heart tissue after MI. TUNEL staining showed greater apoptosis with per2^−/−^ than with WT EPC injection (Fig. 6A and B).

### Per2^−/−^ decreases angiogenesis and arteriogenesis of EPCs after MI

Capillary density in the peri-infarct area was determined by immunohistochemical staining of CD31. Marked angiogenesis was observed in the peri-infarct area in the three groups, but the amount of angiogenesis was greatest with WT EPCs injection (Fig. 6C and D). Many investigators considered that EPCs may contribute to arteriogenesis [33], so we observed the expression of α-SMA as a measure of arteriogenesis in the peri-infarct area. Arteriogenesis was greatest with WT EPC injection (Fig. 6E and F).

## Discussion

Endothelial progenitor cells were identified in 1997 by Asahara *et al*. on the basis of VEGFR2 and CD34 co-expression. Although we lack a clear phenotype of EPCs and their putative precursors and the exact differentiation lineage remains to be determined, it is widely accepted that early EPCs (localized in bone marrow or immediately after migration into the bloodstream) are CD34+/VEGFR2+ cells [34]. This cell population predicts the occurrence of cardiovascular events and death from cardiovascular causes [6,7]. Peripheral blood EPC levels range from 70 to 210 cells/ml [34] to 3000–5000 cells/ml [35], depending likely on the isolation technique used. In our study, we found 1000–2000 EPCs/ml in the bloodstream, which agrees with previous findings. Endothelial progenitor cells have been reported to enhance angiogenesis *in vivo* [36].

Previous studies have shown that mutations of Per2 decrease the production of nitric oxide, vasodilatory prostaglandin(s) [19], myocardial adaptation to ischaemia [37] and mobilization of bone-marrow EPCs [18], all important to cardiovascular disease. Here, we found that Per2 plays an indispensible role in maintaining circulating number and function of EPCs in mice. We found decreased number of circulating EPCs and impaired EPC function in mice with Per2 knockout. We also further provide a possible mechanism by which Per2 affects the function of EPCs. Finally, we observed EPC angiogenesis in a mouse MI model.

Wild-type and per2^−/−^ mice showed some differences in circulating EPC number at different times. Per2 is a key clock gene in regulating the number of circulating EPCs. In previous studies, haematopoietic stem-cell release was found to be decided by core circadian genes (*e.g*. PER1, PER2, Bmal1), which regulate CXC-L12 and stem-cell trafficking indirectly from the central nervous system [17]. However, that study did not investigate how Per2 affects EPC number. In our study, we found that impaired proliferation may contribute to decreased number of circulating EPCs. We also certified the expression of Per2 in cultured bone-marrow EPCs *in vitro*.

Recent studies have shown that impaired function of cells is determined by knockout of Per2 instead of its rhythmic expression [38]. Thus, we compared the function of WT and per2^−/−^ EPCs *in vitro*. Knockout of Per2 impaired proliferation, migration, tube formation and adhesion of EPCs as compared with WT EPCs *in vitro*, which coincides with impaired angiogenesis in mice with MI. Wang *et al*. found that mutated per2 impaired EPC angiogenesis in a mouse hindlimb model, but the mechanisms remained unclear. In our study, we investigated the possible mechanism by which Per2 modulates EPC function. Dysfunctional Per2^−/−^ EPCs were associated with down-regulation of PI3K/Akt/FoxO signalling. Furthermore, PI3K activated by IGF-1 in Per2^−/−^ EPCs blocked the impaired proliferation. PI3k/Akt/FoxO signalling plays an essential role in the proliferation [39] and survival [40,41] of various types of cells, including EPCs. Class IA PI3K comprises heterodimers of a p85 regulatory subunit (p85α, p85β and p55γ) and a p110 catalytic subunit (p110α, p110β and p110γ). In response to stimulation by growth factors, the p110 subunits catalyse the production of a lipid second messenger phosphatidylinositol-3,4,5-trisphosphate at the plasma membrane. This second messenger, in turn, activates the serine/threonine kinase AKT. The catalytic subunits p110α is expressed ubiquitously. Some studies have demonstrated specific roles for p110α in growth factor and insulin signalling [42–44]. The mammalian forkhead box subgroup “O” of forkhead transcription factors consists of FoxO1, FoxO3a, FoxO4 and FoxO6. FoxO proteins are important targets of PI3K/Akt signalling. FoxO3a is phosphorylated by Akt at Thr32, Ser253 and Ser315 [45], which results in decreased FoxO3a DNA-binding activity and/or protein stability. Shukla *et al*. [46] reported that apigenin, by down-regulating Akt–FoxO3a signalling, can inhibit cancer cell proliferation. In our study, decreased activation of PI3k/Akt/FoxO signalling resulted in aberrant proliferation and reduced survival of transplanted EPCs in the ischaemic myocardium.

There are different opinions of the influence of Per2 on Akt signalling. Decreased Akt signalling has been observed in mice with functional disruption of Per2 [47], whereas Wang *et al*. found increased Akt signalling in per2^m/m^ mice. Different results are depended on different tissues and cells.

Knockout of Per2 decreased the expression of CXCR4, which is highly expressed on both endothelial and hematopoietic progenitor cells [48,49]. The SDF1α/CXCR4 cascade is considered important in mobilization, migration and in homing of EPCs [48,49]. However, CXCR4 has an opposite role in mobilization and homing of EPCs [50,51]. Inhibition of CXCR4 with AMD3100 promoted the mobilization of bone-marrow EPCs, but decreased migration of cultured EPCs and impaired the incorporation of EPCs in ischaemia-induced angiogenesis [50]. CXCR4 itself has an important role in mediating the migratory and angiogenic activity of EPC, and CXCR4 contributes to *in vivo* incorporation of EPC and therapeutic neovascularization. Walter *et al*. [51] found that blocking CXCR4 inhibited basal migration as well as VEGF and SDF-1-induced migration; further studies also found that pre-treating of EPCs with anti-CXCR4 antibody significantly reduced recovery of hindlimb blood flow, as well as capillary density. Importantly, incorporation of EPC into ischaemic tissues was significantly lower after pre-incubation of EPCs with the anti-CXCR4 antibody. CXCR4 is the upstream of PI3K/Akt/Foxo signalling pathway. In our study, direct interaction of PER2 and CXCR4 led to reduced expression of CXCR4 by PER2 knockout and impaired migration and homing of EPCs. Activated PI3K signalling in Per2^−/−^ EPCs did not improve CXCR4 expression, which may be the key reason for no improvement in migration, tube formation and adhesion with Per2^−/−^ EPCs. Endothelial progenitor cells contribute to angiogenesis in four steps: mobilize from bone marrow to peripheral blood in response to signals; home to sites of injured tissue repair and angiogenesis; invade and migrate at the same sites; and differentiate into mature ECs [52]. The homing of injected EPCs is most important for treating MI. If homing fails, other functions will not occur. Both PI3K/Akt/FoxO signalling and CXCR4 play important roles in regulating EPC function *in vivo* and *in vitro*. Thus, our findings provided a novel mechanism of Per2 regulating EPC function.

Our *in vivo* studies showed that EPCs contribute to angiogenesis after MI, which is in accordance with previous investigations [10,13,34]. However, angiogenesis was decreased by Per2 knockout. Other investigators found an immune privilege of allogeneic EPCs, so they are useful in therapeutic applications, especially vascular reconstruction [53]. We did not consider immune rejection in our study. Per2 plays an important role in regulating EPC angiogenesis. Less angiogenesis was the outcome of less survival and impaired function of transplanted EPCs, which coincides with decreased cardiac function and increased infarct size after MI.

In conclusion, this is the first study to demonstrate that Per2 is necessary to maintain the number of circulating EPCs and function of bone-marrow EPCs both *in vitro* and *in vivo*. Per2 knockout reduced the number of circulating EPCs and disturbed the function of EPCs. Per2 affected EPC function through CXCR4/PI3k/Akt/FoxO-related mechanisms. Per2 deficiency also impaired EPC survival and angiogenesis in the ischaemic myocardium of mice. Our study independently validated earlier findings of Wang *et al.,* but also extends those findings into the even more clinically relevant model of acute MI and further emphasizes the often overlooked importance of the effect of circadian rhythm genes on cardiovascular disease.

## Supporting information

**Figure S1** (**A**) Real-time RT-PCR analysis of PI3K p110α (NM_008839.2 forward, 5′-CACTCGTCACCATCAAACATGA-3′, and reverse, 5′-AGGGTTGAAAAAGCCGAAGGT-3′). (**B**) PI3K/Akt/FoxO signaling of EPC serum and growth factors starved for 48 hr and the PI3K/Akt/FoxO signaling in bone-marrow mononuclear cells.

## References

[1] Asahara T, Masuda H, Takahashi T (1999). Bone marrow origin of endothelial progenitor cells responsible for postnatal vasculogenesis in physiological and pathological neovascularization. Circ Res.

[2] Rae PC, Kelly RD, Egginton S (2011). Angiogenic potential of endothelial progenitor cells and embryonic stem cells. Vascular Cell.

[3] Takahashi T, Kalka C, Masuda H (1999). Ischemia and cytokine-induced mobilization of bone marrow-derived endothelial progenitor cells for neovascularization. Nat Med.

[4] Walter DH, Rittig K, Bahlmann FH (2002). Statin therapy accelerates reendothelialization: a novel effect involving mobilization and incorporation of bone marrow derived endothelial progenitor cells. Circulation.

[5] Asahara T, Murohara T, Sullivan A (1997). Isolation of putative progenitor endothelial cells for angiogenesis. Science.

[6] Chironi G, Walch L, Pernollet MG (2007). Decreased number of circulating CD34+KDR+ cells in asymptomatic subjects with preclinical atherosclerosis. Atherosclerosis.

[7] Werner N, Kosiol S, Schiegl T (2005). Circulating endothelial progenitor cells and cardiovascular outcomes. N Engl J Med.

[8] Rajasingh J, Thangavel J, Siddiqui MR (2011). Improvement of cardiac function in mouse myocardial infarction after transplantation of epigenetically-modified bone marrow progenitor cells. PLoS ONE.

[9] Park JH, Yoon JY, Ko SM (2011). Endothelial progenitor cell transplantation decreases lymphangiogenesis and adverse myocardial remodeling in a mouse model of acute myocardial infarction. Exp Mol Med.

[10] Iwasaki H, Kawamoto A, Ishikawa M (2006). Dose-dependent contribution of CD34-positive cell transplantation to concurrent vasculogenesis and cardiomyogenesis for functional regenerative recovery after myocardial infarction. Circulation.

[11] Chavakis E, Urbich C, Dimmeler S (2008). Homing and engraftment of progenitor cells: a prerequisite for cell therapy. J Mol Cell Cardiol.

[12] Zheng B, Larkin DW, Albrecht U (1999). The mPer2 gene encodes a functional component of the mammalian circadian clock. Nature.

[13] Sun ZS, Albrecht U, Zhuchenko O (1997). RIGUI, a putative mammalian ortholog of the Drosophila period gene. Cell.

[14] Tsinkalovsky O, Smaaland R, Rosenlund B (2007). Circadian variations in clock gene expression of human bone marrow CD34+ cells. J Biol Rhythms.

[15] Tsinkalovsky O, Filipski E, Rosenlund B (2006). Circadian expression of clock genes in purified hematopoietic stem cells is developmentally regulated in mouse bone marrow. Exp Hematol.

[16] Fukuya H, Emoto N, Nonoka H (2007). Circadian expression of clock genes in human peripheral leukocytes. Biochem Biophys Res Commun.

[17] Méndez-Ferrer S, Lucas D, Battista M (2008). Haematopoietic stem cell release is regulated by circadian oscillations. Nature.

[18] Wang CY, Wen MS, Wang HW (2008). Increased vascular senescence and impaired endothelial progenitor cell function mediated by mutation of circadian gene Per2. Circulation.

[19] Viswambharan H, Carvas JM, Antic V (2007). Mutation of the circadian clock gene Per2 alters vascular endothelial function. Circulation.

[20] Vukolic A, Antic V, Van Vliet BN (2010). Role of mutation of the circadian clock gene Per2 in cardiovascular circadian rhythms. Am J Physiol Regul Integr Comp Physiol.

[21] Voermans C, Kooi ML, Rodenhuis S (2001). *In vitro* migratory capacity of CD34+ cells is related to hematopoietic recovery after autologous stem cell transplantation. Blood.

[22] Britten MB, Abomaali ND, Assmus B (2003). Infarct remodeling following intracoronary progenitor cell treatment in patients with acute myocardial infarction (TOPCARE-AMI): mechanistic insights from serial contrast enhanced magnetic resonance imaging. Circulation.

[23] Krishnamurthy P, Thal M, Verma S (2011). Interleukin-10 deficiency impairs bone marrow-derived endothelial progenitor cell survival and function in ischemic myocardium. Circ Res.

[24] Sumida A, Horiba M, Ishiguro H (2010). Midkine gene transfer after myocardial infarction in rats prevents remodelling and ameliorates cardiac dysfunction. Cardiovasc Res.

[25] Yang Z, Xia WH, Zhang YY (2012). Shear stress-induced activation of Tie2-dependent signaling pathway enhances reendothelialization capacity of early endothelial progenitor cells. J Mol Cell Cardiol.

[26] Zhu JH, Chen JZ, Wang XX (2006). Homocysteine accelerates senescence and reduces proliferation of endothelial progenitor cells. J Mol Cell Cardiol.

[27] Jujo K, Ii M, Losordo DW (2008). Endothelial progenitor cells in neovascularization of infarcted myocardium. J Mol Cell Cardiol.

[28] Su B, Peng X, Jiang D (2013). *In vitro* and *in vivo* evaluations of nano-hydroxyapatite/polyamide 66/glass fibre (n-HA/PA66/GF) as a novel bioactive bone screw. PLoS ONE.

[29] Chapuis N, Tamburini J, Cornillet-Lefebvre P (2010). Autocrine IGF-1/IGF-1R signaling is responsible for constitutive PI3K/Akt activation in acute myeloid leukemia: therapeutic value of neutralizing anti-IGF-1R antibody. Haematologica.

[30] Chen YH, Lin SJ, Lin FY (2007). High glucose impairs early and late endothelial progenitor cells by modifying nitric oxide-related but not oxidative stress-mediated mechanisms. Diabetes.

[31] Huang PH, Chen JS, Tsai HY (2011). Globular adiponectin improves high glucose-suppressed endothelial progenitor cell function through endothelial nitric oxide synthase dependent mechanisms. J Mol Cell Cardiol.

[32] Shen L, Liu Q, Ni J (2009). A proteomic investigation into the human cervical cancer cell line HeLa treated with dicitratoytterbium (III) complex. Chem Biol Interact.

[33] Ruiter MS, van Golde JM, Schaper NC (2010). Diabetes impairs arteriogenesis in the peripheral circulation: review of molecular mechanisms. Clin Sci (Lond).

[34] Asahara T, Kawamoto A (2004). Endothelial progenitor cells for postnatal vasculogenesis. Am J Physiol Cell Physiol.

[35] Peichev M, Naiyer AJ, Pereira D (2000). Expression of VEGFR-2 and AC133 by circulating human CD34(+) cells identifies a population of functional endothelial precursors. Blood.

[36] Kalka C, Masuda H, Takahashi T (2000). Transplantation of ex vivo expanded endothelial progenitor cells for therapeutic neovascularization. Proc Natl Acad Sci.

[37] Eckle T, Hartmann K, Bonney S (2008). Adora2b-elicited Per2 stabilization promotes a HIF-dependent metabolic switch critical for myocardial adaptation to ischemia. Nat Med.

[38] Gu X, Xing L, Shi G (2012). The circadian mutation PER2S662G is linked to cell cycle progression and tumorigenesis. Cell Death Differ.

[39] Heskamp A, Leibinger M, Andreadaki A (2013). CXCL12/SDF-1 facilitates optic nerve regeneration. Neurobiol Dis.

[40] Peng Y, Huang S, Wu Y (2013). Platelet rich plasma clot releasate preconditioning induced PI3K/AKT/NFκB signaling enhances survival and regenerative function of rat bone marrow mesenchymal stem cells in hostile microenvironments. Stem Cells Dev.

[41] Gensch C, Clever Y, Werner C (2007). Regulation of endothelial progenitor cells by prostaglandin E1 *via* inhibition of apoptosis. J Mol Cell Cardiol.

[42] Foukas LC, Claret M, Pearce W (2006). Critical role for the p110alpha phosphoinositide-3-OH kinase in growth and metabolic regulation. Nature.

[43] Knight ZA, Gonzalez B, Feldman ME (2006). A pharmacological map of the PI3-K family defines a role for p110alpha in insulin signaling. Cell.

[44] Zhao JJ, Cheng H, Jia S (2006). The p110alpha isoform of PI3K is essential for proper growth factor signaling and oncogenic transformation. Proc Natl Acad Sci.

[45] Brunet A, Bonni A, Zigmond MJ (1999). Akt promotes cell survival by phosphorylating and inhibiting a Forkhead transcription factor. Cell.

[46] Sanjeev S, Natarajan B, Melissa A (2013). Apigenin inhibits prostate cancer progression in TRAMP mice *via* targeting PI3K/Akt/FoxO pathway. Carcinogenesis.

[47] Carvas JM, Vukolic A, Yepuri G (2012). Period2 gene mutant mice show compromised insulin-mediated endothelial nitric oxide release and altered glucose homeostasis. Front Physiol.

[48] Lapidot T (2001). Mechanism of human stem cell migration and repopulation of NOD/SCID and B2mnull NOD/SCID mice. The role of SDF-1/CXCR4 interactions. Ann N Y Acad Sci.

[49] Rolland-Turner M, Goretti E, Bousquenaud M (2013). Adenosine stimulates the migration of human endothelial progenitor cells. Role of CXCR4 and microRNA-150. PLoS ONE.

[50] Jujo K, Hamada H, Iwakura A (2010). CXCR4 blockade augments bone marrow progenitor cell recruitment to the neovasculature and reduces mortality after myocardial infarction. Proc Natl Acad Sci.

[51] Walter DH, Haendeler J, Reinhold J (2005). Impaired CXCR4 signaling contributes to the reduced neovascularization capacity of endothelial progenitor cells from patients with coronary artery disease. Circ Res.

[52] Caiado F, Dias S (2012). Endothelial progenitor cells and integrins: adhesive needs. Fibrogenesis Tissue Repair.

[53] Ladhoff J, Fleischer B, Hara Y (2010). Immune privilege of endothelial cells differentiated from endothelial progenitor cells. Cardiovasc Res.

