# Supplementary figures and images for: Period 2 is essential to maintain early endothelial progenitor cell function in vitro and angiogenesis after myocardial infarction in mice
Source: J Cell Mol Med. 2014 Mar 13;18(5):907–18. doi: 10.1111/jcmm.12241 (PMC4119396; doi:10.1111/jcmm.12241)

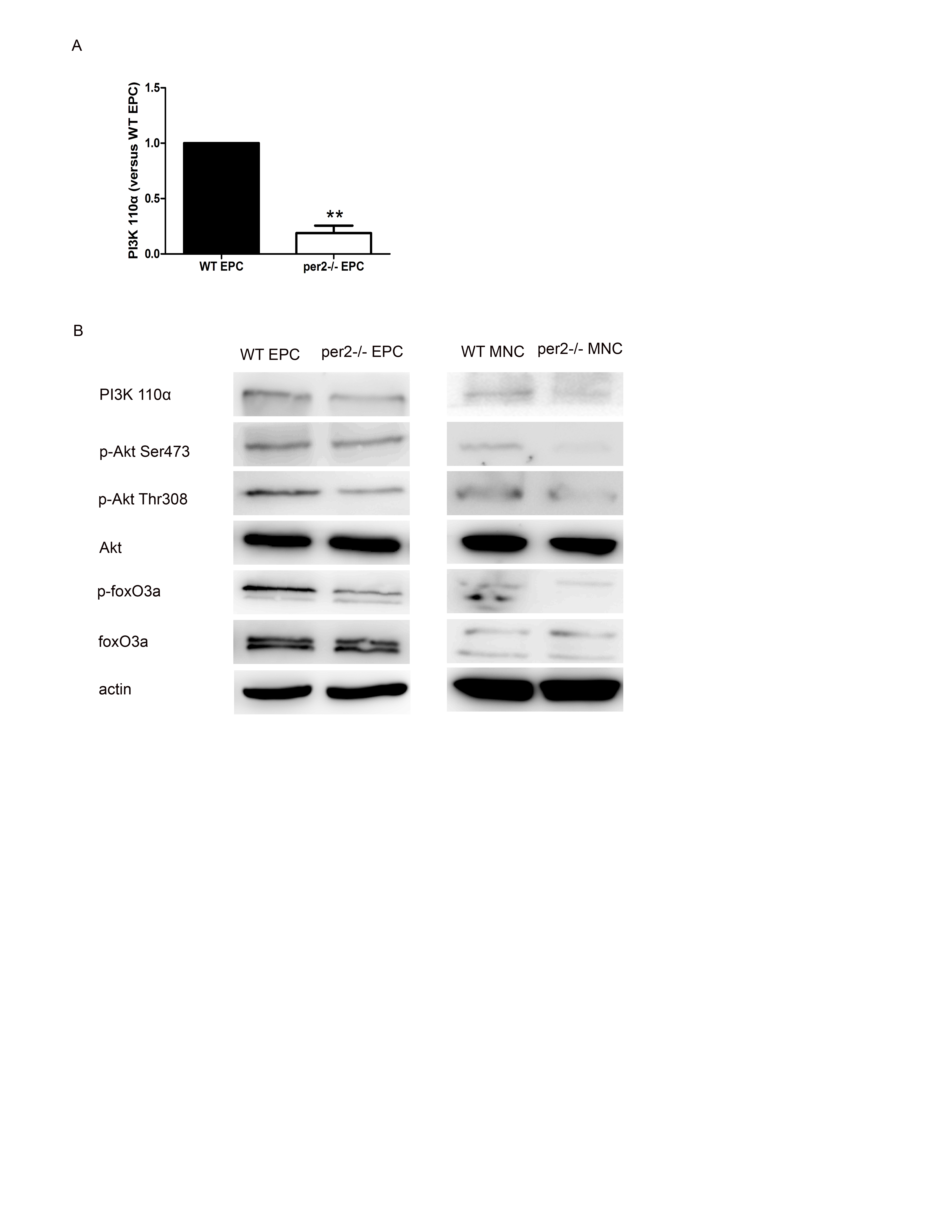

Supplement: Supplementary file 1 — Figure S1 (A) Real-time RT-PCR analysis of PI3K p110α (NM_008839.2 forward, 5′-CACTCGTCACCATCAAACATGA-3′, and reverse, 5′-AGGGTTGAAAAAGCCGAAGGT-3′). (B) PI3K/Akt/FoxO signaling of EPC serum and growth factors starved for 48 hr and the PI3K/Akt/FoxO signaling in bone-marrow mononuclear cells. [file jcmm0018-0907-SD1.tif]
